# Key parameters in intratumoral-peritumoral region fusion models: optimizing deep learning radiomics for breast cancer diagnosis

**DOI:** 10.3389/fonc.2025.1587879

**Published:** 2025-09-03

**Authors:** Jun-Tao Shen, Gong-Quan Chen, Hai-Mei Lun, Hua-Fang Huang, Ling Zhang, Ling-Ling Li, Yun-Xia Deng, Hui-Hua Wu, Qiao Hu

**Affiliations:** ^1^ Guangxi Medical University, Nanning, Guangxi, China; ^2^ Department of Ultrasound, People’s Hospital of Guangxi Zhuang Autonomous Region & Guangxi Academy of Medical Sciences, Nanning, Guangxi, China; ^3^ Department of Ultrasound, Minda Hospital of Hubei Minzu University, Enshi, Hubei, China; ^4^ Department of Breast Surgery, Guilin Municipal Hospital of Traditional Chinese Medicine, Guilin, Guangxi, China; ^5^ Department of Ultrasound, Fangchenggang First People’s Hospital, Fangchenggang, Guangxi, China; ^6^ Guilin Medical University, Guilin, Guangxi, China

**Keywords:** deep learning radiomics, multicenter, breast cancer, peritumoral region, contrast-enhanced ultrasound

## Abstract

**Background:**

Early diagnosis of breast cancer (BC) is crucial for improving patient outcomes. Features of the peritumoral region have been shown to significantly enhance the predictive performance of deep learning radiomics (DLR) models. This study aims to explore the impact of key parameter selection on improving the performance of the intratumoral-peritumoral region fusion model. The goal is to enhance the modal’s non-invasive diagnostic capability for distinguishing benign and malignant breast tumors.

**Materials and methods:**

This retrospective study included 411 female patients with breast lesions from four hospitals. DLR models were constructed using their contrast-enhanced ultrasound (CEUS) images. The intratumoral region of interest (ROI) was gradually expanded to generate peritumoral regions of varying thicknesses. Six groups of fusion models were constructed using different key parameter combinations, including pseudo-color (PC) vs. grayscale (GRAY) images, original precise (OP) ROI vs. bounding box (BB) ROI, and direct extension (DE) strategy vs. feature-level fusion (FLF) strategy. Additionally, a reader study was conducted, comparing the diagnostic performance of the best fusion model with that of six radiologists. The performance of the models was evaluated using the area under the curve (AUC).

**Results:**

Incorporating the peritumoral region significantly enhanced the diagnostic performance of the DLR models. The PC-OP-DE-Peri (4mm) model achieved the highest performance in the testing cohort, with an AUC of 0.837. The performance surpassed both the intratumoral models and all radiologists. The effects of different key parameter selections on fusion model performance varied.

**Conclusion:**

This study suggests that the selection of PC images, OP ROIs, and the DE strategy effectively improves the performance of intratumoral-peritumoral region fusion models for predicting BC.

## Introduction

Deep learning radiomics (DLR), an emerging field, has garnered considerable attention in the medical community in recent years. By extracting high-throughput image features, DLR provides non-invasive biomarkers for clinical outcomes. Unlike traditional radiomics, DLR can learn directly from raw images and automatically extract appropriate, undefined features (1, 2). DLR plays a significant role in lesion detection and cancer diagnosis (3). In the context of personalized medicine, DLR holds promise for significant improvements in disease diagnosis, prognosis evaluation, and treatment response prediction.

Breast cancer (BC) is the most common malignancy in women worldwide, with 2.31 million new cases reported in 2022 (4). The development of advanced imaging techniques, such as DLR, has been pivotal in improving the diagnosis and treatment of BC (5). Recent studies have demonstrated that DLR based on ultrasound images can effectively predict various critical clinical outcomes in BC, such as response to neoadjuvant chemotherapy (6, 7), sentinel lymph node metastasis (8), axillary lymph node metastasis (9), tumor benignity or malignancy (10, 11), disease-free survival (12), molecular subtypes (13), and recurrence (14). Furthermore, contrast-enhanced ultrasound (CEUS) provides significant advantages in assessing tumor blood flow and microvascular status. These benefits enhanced the accuracy of BC diagnosis (15). Recent imaging advances, like hyperspectral imaging–based computer-aided detection (16), have improved lesion detection but face challenges in clinical use due to high costs and complexity. Socioeconomic disparities limit screening, with family support and economic status as key factors for early diagnosis (17). These challenges highlight the need for diagnostic methods that are accurate, affordable, interpretable, and widely usable. Thus, developing a CEUS-based DLR model to classify breast tumors as benign or malignant is highly promising.

Although many existing DLR studies focus on the intratumoral region (18, 19), increasing evidence indicates that the peritumoral region can also offer valuable supplementary information (20, 21). Recent studies have shown that changes in the tissue surrounding the tumor, including angiogenic factors (22), lymphangiogenesis (23), peritumoral lymphocytic infiltration (24), peripheral edema (25), and stromal response (26), are important indicators of clinical outcomes. Thus, the peritumoral region may provide valuable information for tumor diagnosis and prognosis. Recent studies have extensively utilized the characteristics of the peritumoral region. Sun et al. (20) utilized peritumoral features to predict axillary lymph node metastasis in BC. Liu et al. (27) evaluated lymphatic vessel invasion in BC using peritumoral features. These studies further validate the feasibility of constructing a DLR intratumoral-peritumoral region fusion model to predict the benignity or malignancy of breast tumors.

Despite numerous studies attempting to integrate peritumoral region features, significant discrepancies remain in the key parameters for constructing intratumoral-peritumoral region fusion models. In terms of image color selection, some studies retain the pseudo-color (PC) of the image (28), while others convert the image to grayscale (GRAY) (29). Regarding region of interest (ROI) shape selection, some studies directly use the original precise (OP) ROI for image analysis (30). In contrast, others employ bounding box (BB) ROIs, which expand outward from the OP ROI to form the smallest enclosing rectangle (31). Concerning the fusion strategy of intratumoral and peritumoral regions, some studies adopt a direct extension (DE) strategy. In this approach, the intratumoral region is directly expanded to obtain the peritumoral region, and features are extracted from the entire region (32). In contrast, other studies employ a feature-level fusion (FLF) strategy. In the FLF approach, features are separately extracted from the intratumoral and peritumoral regions and then fused to construct the model (33). Although these strategies have achieved some success, there is currently a lack of systematic research exploring how to determine the optimal parameters. This uncertainty hinders the widespread clinical application of these advancements.

This study aims to identify the optimal parameters for constructing an intratumoral-peritumoral fusion model using DLR and CEUS images. Specifically, we focus on evaluating how different combinations of these parameters affect diagnostic performance. The goal is to enhance the ability of DLR models to predict the benign or malignant nature of breast tumors. Specifically, this study will address the following three core issues: (1) Comparison of CEUS image color selection (PC vs. GRAY); (2) Selection of ROI shape (OP ROI vs. BB ROI); (3) Selection of fusion strategy for intratumoral and peritumoral regions (DE strategy vs. FLF strategy).

## Materials and methods

### Patients

This study is a retrospective multicenter study that included 411 female patients with breast lesions. The study was conducted at four hospitals in different regions of China, with data collected between January 2018 and May 2024. A total of 307 patients were from People’s Hospital of Guangxi Zhuang Autonomous Region (Hospital 1), 30 from Guilin Municipal Hospital of Traditional Chinese Medicine (Hospital 2), 28 from Fangchenggang First People’s Hospital (Hospital 3), and 46 from Minda Hospital of Hubei Minzu University (Hospital 4). To minimize selection bias, we enrolled consecutive patients from four hospitals with diverse imaging protocols and settings. Only non-diagnostic images were excluded. No manual balancing by lesion type was done to preserve real-world diversity. The study was conducted in accordance with the Declaration of Helsinki and approved by the ethics committees of all participating hospitals. Due to the retrospective design, patient consent was not required for the study.

All lesions were confirmed by pathology based on ultrasound-guided core-needle biopsy or surgical excision. Pathological diagnoses were made following the WHO Classification of Breast Tumors (5th edition) and institutional protocols, performed by board-certified breast pathologists.

Inclusion criteria were: (1) pathologically confirmed breast lesions; (2) standard and complete CEUS examination performed; (3) imaging examinations met quality standards. Exclusion criteria were: (1) incomplete imaging or clinical data; (2) patients who had received chemotherapy, radiotherapy, or targeted therapy; (3) pregnant or lactating patients at the time of imaging examination.

**Figure 1:**
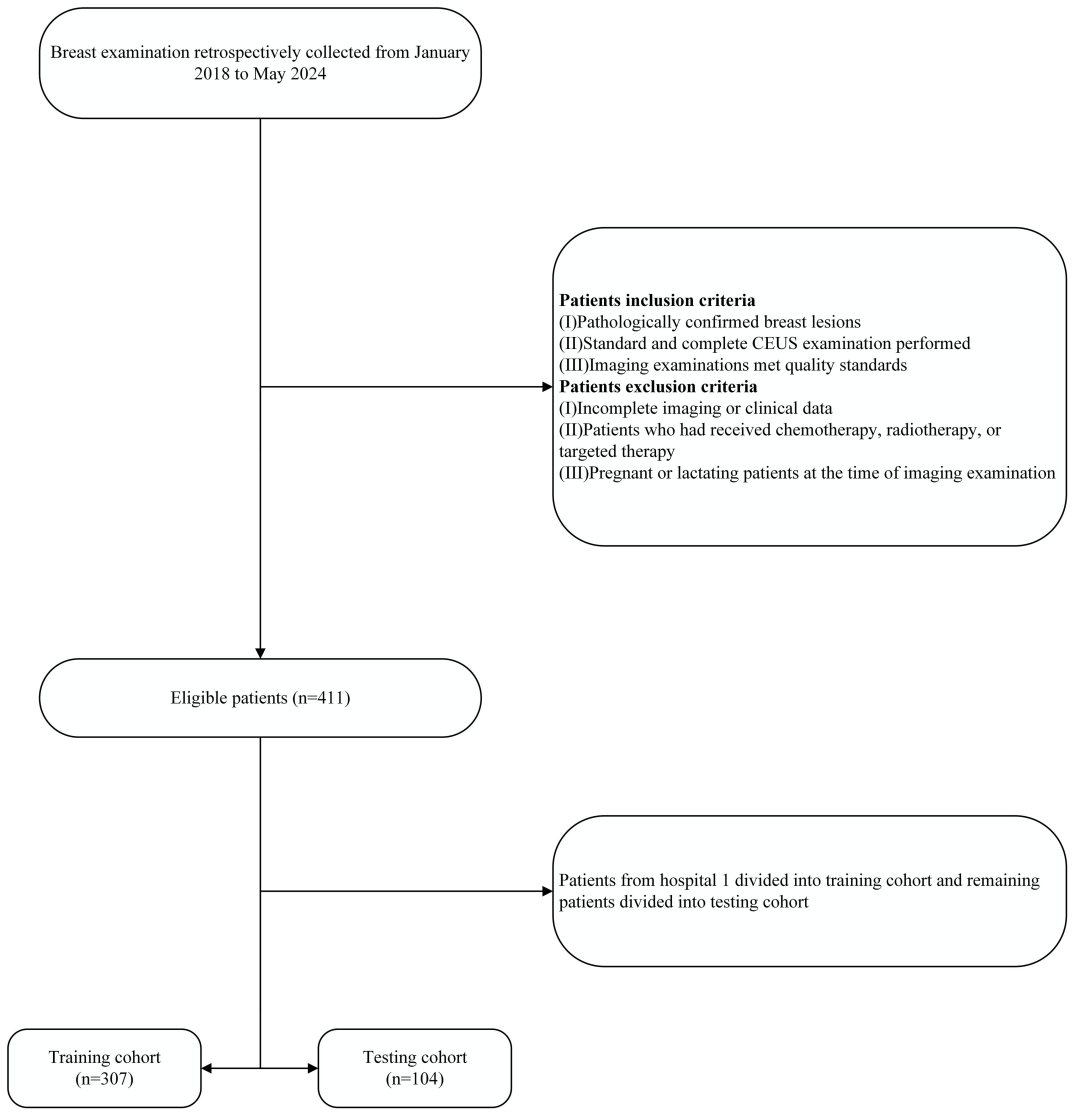
The patient recruitment flowchart for the present study. CEUS, contrast-enhanced ultrasound.

To reduce overfitting and potential bias, a center-split cohort strategy was adopted. Data from Hospital 1, the largest patient group (n = 307), were used as the training cohort, while data from Hospitals 2, 3, and 4 (n = 104) formed the testing cohort. The testing data were obtained from independent external centers to mimic real-world generalization. This approach also helps minimize center-specific bias and is consistent with previous multicenter radiomics studies (34–36). Detailed information on the patient selection process is shown in **Figure 1**, and clinical baseline characteristics were extracted from the databases of each hospital.

### Acquisition of CEUS images

Ultrasound examinations were performed using equipment including Aplio 500, Aplio i800, Aplio i900, Mindray R7, Mindray R9, GE LOGIQ E9, and GE LOGIQ E10, all equipped with high-frequency linear array probes. To reduce inter-machine variability, all CEUS scans were performed by radiologists from the same department (≥5 years’ experience) using a standardized protocol. Device settings including gain, depth, mechanical index, and focal length were unified before scanning. All images were reviewed by a senior breast imaging specialist (>20 years’ experience) to ensure quality, as recommended by prior multi-device imaging studies (29). During the examination, patients were placed in the supine position with both arms raised to ensure optimal exposure for breast imaging. Suspicious lesions were scanned in multiple transverse views to assess their size, location, and other morphological characteristics. The slice showing the largest lesion was then selected for CEUS imaging. In CEUS mode, 4.8 mL of contrast agent (SonoVue) was injected via the median cubital vein, followed by a flush with 5 mL of saline. The time storage functions were activated, and dynamic images were recorded for a minimum of 2 minutes. All ultrasound images were stored in DICOM format on the workstation for further analysis.

### ROI segmentation and processing

In CEUS imaging, the peak phase with the highest enhancement intensity was determined using the time-intensity curve (TIC), based on the largest tumor section. The single frame corresponding to the peak enhancement moment was selected as the representative. This method relies on the significant difference in peak enhancement between benign and malignant tumor masses (37). CEUS images were exported from the workstation in JPG format and processed using ITK-SNAP software (http://www.itksnap.org). A radiologist with over ten years of experience in breast ultrasound examination independently identified the ROI. The ROI was then reviewed and optimized by another radiologist with 20 years of diagnostic experience to ensure consistency and accuracy.

**Figure 2:**
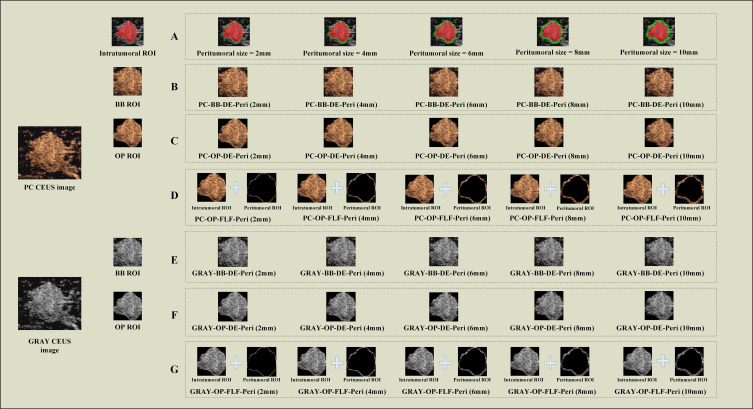
The representative images for DLR model construction. **(A)** Intratumoral ROIs (red) and peritumoral ROIs of different sizes (green). **(B)** The schematic images of PC-BB-DE strategy. **(C)** The schematic images of PC–OP-DE strategy; **(D)** The schematic images of PC-OP-FLF; **(E)** The schematic images of GRAY-BB-FLF strategy; **(F)** The schematic images of GRAY-OP-DE strategy; **(G)** The schematic images of GRAY-OP-FLF strategy. PC, pseudo-color; BB, bounding box; Intra, intratumoral regions; OP, original precise; GRAY, grayscale; DE, direct extension; Peri, peritumoral regions; FLF, feature-level fusion; DLR, deep learning radiomics; CEUS, contrast-enhanced ultrasound; ROI, region of interest.

Using the mask padding toolkit from the OnekeyAI platform (https://github.com/onekeyai-platform/onekey), we gradually expanded the intratumoral ROI. The expansion was performed in 2mm increments in each direction up to 10mm. This approach followed the strategy of Ding et al. (38), who showed that stepwise peritumoral region expansion is feasible and useful in radiomics analysis for BC. The toolkit is implemented using the “SimpleItk” package in Python version 3.7. Ultimately, five peritumoral regions were obtained: 2mm, 4mm, 6mm, 8mm, and 10mm. Both the intratumoral and peritumoral regions were used for further analysis. **Figure 2** illustrates the process of expanding the intratumoral region.

### Different parameters for constructing intratumoral-peritumoral region fusion models

Three factors were considered to determine the optimal parameters: (1) CEUS image color selection: the images were divided into two groups, the PC image group (retaining color) and the GRAY image group (converted to grayscale). (2) ROI shape selection: the images were categorized into the OP ROI group and the BB ROI group based on the shape of the ROI. The OP ROI retains only the delineated ROI area, with background pixels outside the ROI removed. In contrast, the BB ROI retains the smallest enclosing rectangle containing the OP ROI. (3) Fusion strategy selection: regarding the fusion strategy for intratumoral and peritumoral regions, the images were categorized into the FLF group and the DE group. FLF involves feature-level fusion, where features are first extracted from the intratumoral and peritumoral ROIs and then combined. DE involves expanding the intratumoral ROI to create a new ROI that includes both intratumoral and peritumoral regions. Features are then directly extracted from this combined ROI. Based on the above strategies, this study constructed six groups of different model combinations to evaluate the impact of each parameter on model performance: (1) PC-BB-DE; (2) GRAY-BB-DE; (3) PC-OP-DE; (4) GRAY-OP-DE; (5) PC-OP-FLF; and (6) GRAY-OP-FLF. The combinations ‘PC-BB-FLF’ and ‘GRAY-BB-FLF’ were excluded due to the partial overlap between the intratumoral BB ROI and peritumoral ROI, which could affect model accuracy. **Figure 2** illustrates the entire process of constructing the intratumoral-peritumoral region fusion model. To enhance clarity, **Table 1** provides clear definitions of abbreviations used in the model names, along with representative examples.

**Table 1:** Abbreviations and descriptions of model naming components.

Abbreviation or example	Full term/Interpretation	Description
PC	Pseudo-Color	CEUS image preserving original color information.
GRAY	Grayscale	CEUS image converted to grayscale
OP	Original Precise	Precisely delineates the tumor region, retains only ROI pixels, and removes background interference.
BB	Bounding Box	Minimum enclosing rectangle of the tumor, focusing on the lesion’s core area.
DE	Direct Extension	Expands the intratumoral ROI outward to include the peritumoral region; features are extracted from the entire expanded area.
FLF	Feature-Level Fusion	Intratumoral and peritumoral features are separately extracted and then fused at the feature level.
Peri (X mm)	Peritumoral Region with X mm Extension	Region defined by radial expansion from the tumor boundary (e.g., 2mm, 4mm, 6mm).
PC-OP-DE-Peri (4mm)	Pseudo-Color Image + Original Precise ROI + Direct Extension Strategy + 4mm Peritumoral Region	Example of a fusion model that preserves CEUS color information, keeps only ROI pixels, expands 4mm radially around the tumor, and directly extracts features from the expanded image.
GRAY-BB-FLF-Peri (6mm)	Grayscale Image + Bounding Box ROI + Feature-Level Fusion + 6mm Peritumoral Region	Example of a fusion model that converts CEUS images to grayscale, uses the bounding box of the OP ROI, expands 6mm radially, extracts intratumoral and peritumoral features separately, and fuses them at the feature level.

PC, pseudo-color; CEUS, contrast-enhanced ultrasound; GRAY, grayscale; OP, original precise; BB, bounding box; DE, direct extension; FLF, feature-level fusion; Peri, peritumoral regions; ROI, region of interest.

### DLR feature extraction and model construction

All DLR analyses were performed using the OnekeyAI platform (version 4.9.1). This Python-based system includes popular libraries such as PyTorch (1.11.0), CUDA (11.3.1), cuDNN (8.2.1), and Scikit-learn (1.0.2). All input images were resized to a uniform size of 224 × 224 pixels. First, the widely used DLR model VGG16 was pre-trained on the ImageNet dataset, and transfer learning was applied to the training cohort. After training the VGG16 model, deep features were extracted from the fifth-to-last pooling layer (block1_pool). This layer preserves more low-level spatial details. A total of 100,352 features (112 × 112 × 8) were obtained. This layer was chosen instead of the final average pooling layer because it performed better on our dataset. Principal Component Analysis (PCA) was applied to reduce the dimensionality of the DLR features to 32, enhancing the model’s generalizability and reducing the risk of overfitting. Feature selection was performed using the Mann-Whitney U test, retaining features with a *p*-value less than 0.05. Next, Pearson correlation coefficients were calculated to assess feature redundancy, and redundant features with an absolute correlation coefficient of 0.9 or greater were removed. Feature selection was performed using Least Absolute Shrinkage and Selection Operator (LASSO) regression, and the most representative features were selected via ten-fold cross-validation.

To select the optimal classifier for downstream prediction, we used the built-in Scikit-learn (version 1.0.2) module in the Onekey AI platform (version 4.9.1). This platform includes several commonly used machine learning algorithms, such as Support Vector Machine (SVM), Random Forest (RF), XGBoost, and Logistic Regression (LR). We compared the classification performance of these algorithms during model selection. LR model showed the highest and most consistent performance across multiple metrics, including area under the curve (AUC), accuracy, specificity, and sensitivity. Therefore, we selected LR model as the final classifier for all reported results. Although LR is a linear model, we believe that the deep convolutional layers in the feature extraction network captured enough nonlinear and abstract patterns. This allowed the linear classifier to perform well without increasing the risk of overfitting. This approach also improves model transparency and reproducibility, which are essential for clinical decision support.

**Figure 3:**
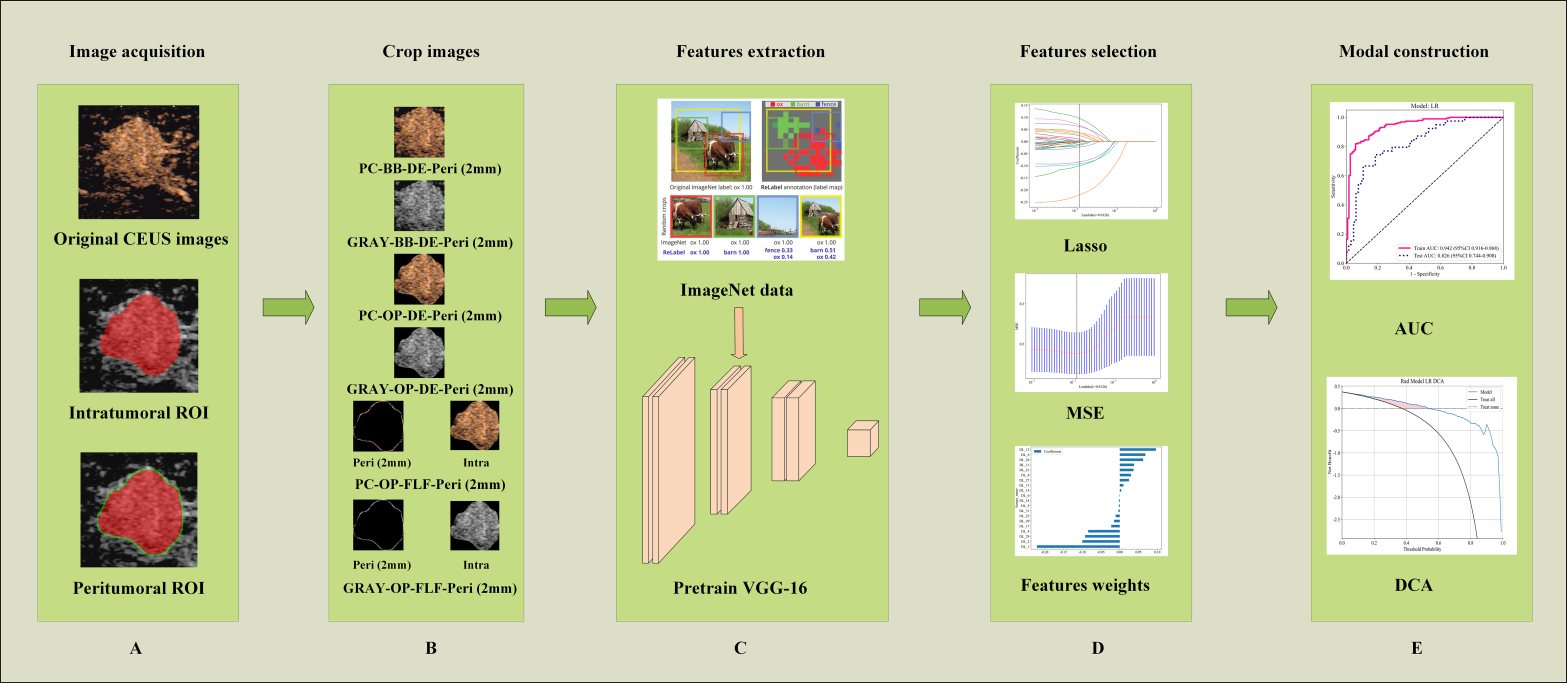
Workflow of ROI extraction and DLR modal construction. **(A)** Illustrating the image segmentation process, where the red area denotes the intratumoral ROI and the green area represents the peritumoral ROI. **(B)** An illustration of obtaining different ROI crops. **(C)** The process of DLR feature extraction. **(D)** The feature selection processing. **(E)** The model performance evaluation. AUC, the area under the curve; PC, pseudo-color; BB, bounding box; Intra, intratumoral regions; OP, original precise; GRAY, grayscale; DE, direct extension; Peri, peritumoral regions; ROI, region of interest; DLR, deep learning radiomics; CEUS, contrast-enhanced ultrasound.

The selected features were input into an LR model to build the radiomics model. Five-fold cross-validation was performed to validate the reliability of the selected features. After training, the model’s predictive performance was evaluated on both the training and testing cohorts. Evaluation metrics included the receiver operating characteristic (ROC) curve, AUC, accuracy, sensitivity, and specificity. The model construction process is illustrated in **Figure 3**.

To enhance model interpretability, Gradient-weighted Class Activation Mapping (Grad-CAM) was applied to produce heatmaps showing image areas that most influence classification.

### A reader study

A reader study was conducted to compare the diagnostic performance of DLR models and radiologists. Six radiologists participated, including two junior radiologists with less than five years of experience, two intermediate radiologists with five to ten years of experience, and two senior radiologists with more than ten years of experience. The testing cohort consisted of 104 breast tumor cases, which were presented to the radiologists in a random order. Throughout the study, radiologists were blinded to the original diagnostic reports, peer opinions, and final pathological results to ensure an unbiased evaluation process.

### Statistics

All data analyses were conducted on the OnekeyAI platform (version 4.9.1) using Python 3.7.12. Statistical analyses were performed using Statsmodels (version 0.13.2). The LR model was implemented using Scikit-learn (version 1.0.2). The DLR framework was developed using PyTorch (version 1.11.0) and optimized with CUDA (version 11.3.1) and cuDNN (version 8.2.1). In clinical data analysis, continuous variables were assessed with the Mann-Whitney U test or independent t-test, while categorical variables were analyzed using the chi-square test or Fisher’s exact test. A result was considered statistically significant if the two-sided *p*-value was less than 0.05.

## Results

### Clinical characteristics

This study included 411 female patients with breast lesions from four different hospitals. Of these, 227 were diagnosed as malignant and 184 as benign based on pathological evaluations. The clinical characteristics of the patients are summarized in **Table 2**.

**Table 2:** Characteristics of patients in the training and testing cohort.

Characteristics	Training cohort (n = 307)			Testing cohort (n = 104)		
	Benign	Malignancy	*P*	Benign	Malignancy	*P*
Age (years)	43.11 ± 11.61	51.86 ± 11.42	<0.001	43.45 ± 11.50	48.82 ± 9.66	0.005
Size (mm)	18.02 ± 11.23	25.51 ± 11.31	<0.001	13.26 ± 8.60	17.21 ± 12.24	0.014
BI-RADS-category			<0.001			<0.001
3	31(26.05)	2(1.06)		11(16.92)	1(2.56)	
4A	49(41.18)	3(1.60)		45(69.23)	15(38.46)	
4B	27(22.69)	19(10.11)		8(12.31)	9(23.08)	
4C	12(10.08)	60(31.91)		1(1.54)	9(23.08)	
5	null	104(55.32)		null	5(12.82)	
Location			0.082			0.847
UOQ	50(42.02)	107(56.91)		34(52.31)	18(46.15)	
LOQ	27(22.69)	29(15.43)		11(16.92)	6(15.38)	
LIQ	12(10.08)	14(7.45)		8(12.31)	7(17.95)	
UIQ	30(25.21)	38(20.21)		12(18.46)	8(20.51)	
Orientation			0.008			1.0
Parallel	114(95.80)	161(85.64)		57(87.69)	34(87.18)	
Not parallel	5(4.20)	27(14.36)		8(12.31)	5(12.82)	
Enhancement intensity			<0.001			<0.001
Iso or hypo	77(64.71)	22(11.70)		42(64.62)	7(17.95)	
Hyper	42(35.29)	166(88.30)		23(35.38)	32(82.05)	
Enhancement homogeneity			<0.001			0.002
Homogenous	80(67.23)	46(24.47)		42(64.62)	12(30.77)	
Heterogeneous	39(32.77)	142(75.53)		23(35.38)	27(69.23)	
Enhancement scope			<0.001			<0.001
Not enlarged	99(83.19)	51(27.13)		52(80.00)	12(30.77)	
Enlarged	20(16.81)	137(72.87)		13(20.00)	27(69.23)	
CEUS margin			<0.001			<0.001
Clear	94(78.99)	40(21.28)		48(73.85)	13(33.33)	
Obscure	25(21.01)	148(78.72)		17(26.15)	26(66.67)	
Perfusion defect			<0.001			0.037
Absent	106(89.08)	132(70.21)		52(80.00)	23(58.97)	
Present	13(10.92)	56(29.79)		13(20.00)	16(41.03)	
Crab claw-like sign			<0.001			0.005
Absent	116(97.48)	158(84.04)		62(95.38)	29(74.36)	
Present	3(2.52)	30(15.96)		3(4.62)	10(25.64)	

UOQ, upper outer quadrant; LOQ, lower outer quadrant; UIQ, upper inner quadrant; LIQ, lower inner quadrant; CEUS, contrast-enhanced ultrasound.

### Intratumoral region models performance analysis

In the testing cohort, the AUC of the PC-BB-Intra model was 0.801, while that of the GRAY-BB-Intra model was 0.792. The AUC of the PC-OP-Intra model was 0.807, while that of the GRAY-OP-Intra model was 0.802. The intratumoral model using PC images and the OP ROI demonstrated the best diagnostic performance in the testing cohort. Retaining the PC images or choosing the OP ROI improved model performance under otherwise identical conditions (**Table 3**).

**Table 3:** The comparison of the diagnostic performance between different intratumoral and intratumoral-peritumoral region fusion models.

Model	Cohort	AUC	Accuracy	Sensitivity	Specificity	Cohort	AUC	Accuracy	Sensitivity	Specificity
PC-BB-Intra	Training	0.941	0.863	0.856	0.874	Testing	0.801	0.740	0.821	0.692
PC-OP-Intra	Training	0.908	0.837	0.846	0.824	Testing	0.807	0.740	0.744	0.738
GRAY-BB-Intra	Training	0.920	0.857	0.846	0.874	Testing	0.792	0.760	0.769	0.754
GRAY-OP-Intra	Training	0.871	0.805	0.803	0.807	Testing	0.802	0.750	0.692	0.785
PC-BB-DE-Peri (2mm)	Training	0.942	0.863	0.814	0.941	Testing	0.826	0.798	0.641	0.892
PC-OP-DE-Peri (2mm)	Training	0.908	0.821	0.814	0.832	Testing	0.827	0.808	0.641	0.908
PC-OP-FLF-Peri (2mm)	Training	0.947	0.879	0.899	0.849	Testing	0.813	0.769	0.718	0.800
GRAY-BB-DE-Peri (2mm)	Training	0.893	0.834	0.856	0.798	Testing	0.814	0.788	0.692	0.846
GRAY-OP-DE-Peri (2mm)	Training	0.874	0.811	0.761	0.891	Testing	0.826	0.769	0.769	0.769
GRAY-OP-FLF-Peri (2mm)	Training	0.921	0.827	0.777	0.908	Testing	0.807	0.769	0.564	0.892
PC-BB-DE-Peri (4mm)	Training	0.943	0.870	0.862	0.882	Testing	0.814	0.817	0.692	0.892
PC-OP-DE-Peri (4mm)	Training	0.900	0.831	0.867	0.773	Testing	0.837	0.808	0.667	0.892
PC-OP-FLF-Peri (4mm)	Training	0.943	0.870	0.856	0.891	Testing	0.817	0.750	0.795	0.723
GRAY-BB-DE-Peri (4mm)	Training	0.919	0.863	0.856	0.874	Testing	0.804	0.760	0.846	0.708
GRAY-OP-DE-Peri (4mm)	Training	0.876	0.814	0.856	0.748	Testing	0.834	0.731	0.872	0.646
GRAY-OP-FLF-Peri (4mm)	Training	0.913	0.834	0.824	0.849	Testing	0.808	0.750	0.615	0.831
PC-BB-DE-Peri (6mm)	Training	0.938	0.873	0.867	0.882	Testing	0.777	0.779	0.692	0.831
PC-OP-DE-Peri (6mm)	Training	0.859	0.782	0.755	0.824	Testing	0.826	0.760	0.718	0.785
PC-OP-FLF-Peri (6mm)	Training	0.967	0.906	0.904	0.908	Testing	0.806	0.731	0.821	0.677
GRAY-BB-DE-Peri (6mm)	Training	0.914	0.821	0.755	0.924	Testing	0.757	0.731	0.615	0.800
GRAY-OP-DE-Peri (6mm)	Training	0.879	0.821	0.830	0.807	Testing	0.815	0.750	0.821	0.708
GRAY-OP-FLF-Peri (6mm)	Training	0.920	0.860	0.878	0.832	Testing	0.786	0.702	0.718	0.692
PC-BB-DE-Peri (8mm)	Training	0.940	0.876	0.899	0.840	Testing	0.765	0.683	0.846	0.585
PC-OP-DE-Peri (8mm)	Training	0.863	0.811	0.840	0.765	Testing	0.781	0.683	0.795	0.615
PC-OP-FLF-Peri (8mm)	Training	0.961	0.889	0.872	0.916	Testing	0.770	0.712	0.897	0.600
GRAY-BB-DE-Peri (8mm)	Training	0.905	0.824	0.771	0.908	Testing	0.763	0.712	0.769	0.677
GRAY-OP-DE-Peri (8mm)	Training	0.873	0.811	0.771	0.874	Testing	0.766	0.702	0.769	0.662
GRAY-OP-FLF-Peri (8mm)	Training	0.910	0.844	0.819	0.882	Testing	0.760	0.663	0.769	0.600
PC-BB-DE-Peri (10mm)	Training	0.945	0.893	0.883	0.908	Testing	0.795	0.731	0.872	0.646
PC-OP-DE-Peri (10mm)	Training	0.903	0.821	0.819	0.824	Testing	0.804	0.769	0.641	0.846
PC-OP-FLF-Peri (10mm)	Training	0.968	0.912	0.920	0.899	Testing	0.781	0.692	0.949	0.538
GRAY-BB-DE-Peri (10mm)	Training	0.914	0.847	0.824	0.882	Testing	0.774	0.740	0.769	0.723
GRAY-OP-DE-Peri (10mm)	Training	0.894	0.837	0.899	0.739	Testing	0.780	0.731	0.795	0.692
GRAY-OP-FLF-Peri (10mm)	Training	0.909	0.824	0.830	0.815	Testing	0.777	0.692	0.744	0.662

AUC, the area under the curve; PC, pseudo-color; BB, bounding box; Intra, intratumoral regions; OP, original precise; GRAY, grayscale; DE, direct extension; Peri, peritumoral regions; FLF, feature-level fusion.

### Intratumoral-peritumoral region fusion models performance analysis

**Figure 4:**
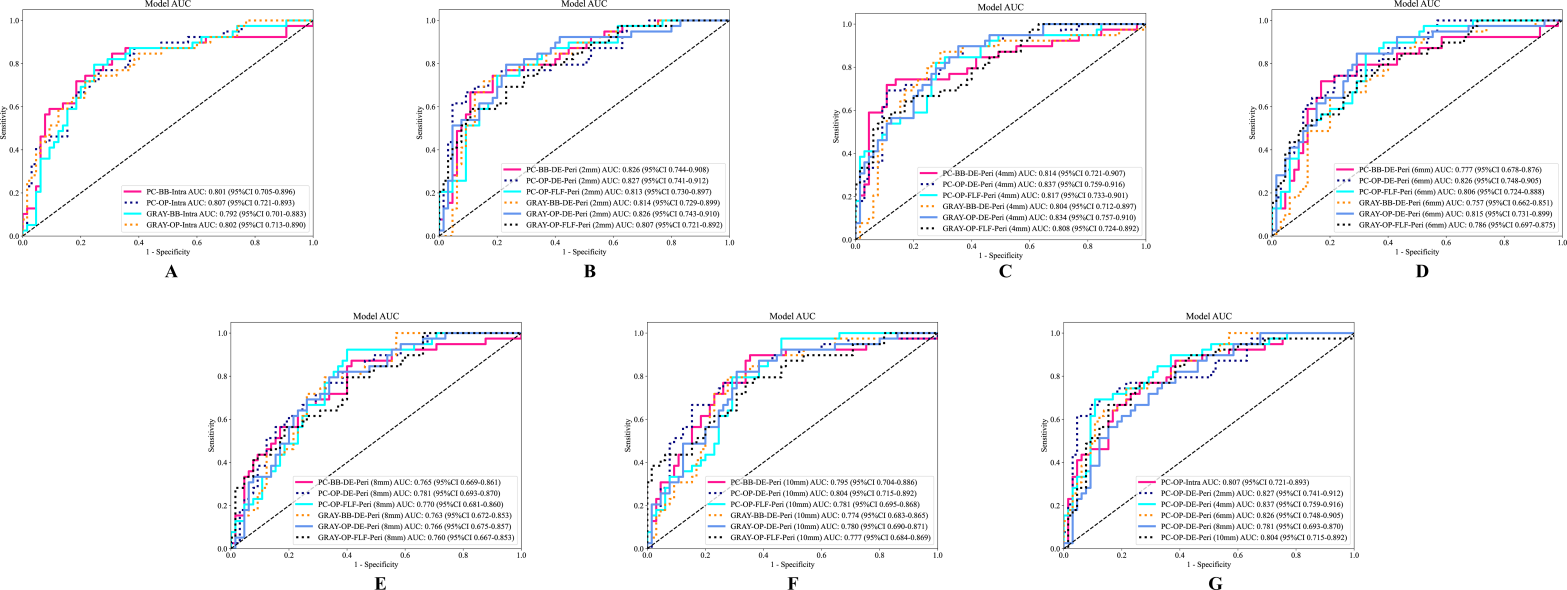
The ROC curves of different models in the testing cohort. **(A)** The ROC curve of the intratumoral model. **(B–F)** Display the ROC curves of fusion models with peritumoral regions extended to 2mm, 4mm, 6mm, 8mm, and 10mm, respectively. **(G)** The ROC curves of the optimal intratumoral model and the optimal extending different peritumoral sizes fusion models. AUC, the area under the curve; ROC, receiver operating characteristic; PC, pseudo-color; BB, bounding box; Intra, intratumoral regions; OP, original precise; GRAY, grayscale; DE, direct extension; Peri, peritumoral regions; DLR, deep learning radiomics.

This study constructed six groups of intratumoral-peritumoral region fusion models. The performance metrics, including AUC, accuracy, sensitivity, and specificity for all models in training and testing cohorts, are summarized in **Table 3**. Incorporating peritumoral information significantly enhanced model performance. Extending a certain peritumoral region enhanced the model’s diagnostic capability among the six model construction strategies. Compared to the best-performing intratumoral region model (AUC=0.807), most intratumoral-peritumoral region fusion models with 2mm and 4mm extensions showed improved diagnostic performance. These results suggest that the inclusion of peritumoral regions enhances model performance. However, models extending 6mm, 8mm, and 10mm generally exhibited lower diagnostic performance than the intratumoral region model. These results suggest that the over-expansion of the peritumoral region leads to a decline in performance. **Table 4** summarizes the differences in AUC, sensitivity, specificity, and accuracy between the fusion models and the best intratumoral model. **Figure 4** shows the AUC of all models in the testing cohort.

**Table 4:** Relative performance (Δ) of intratumoral–peritumoral fusion models compared with the best intratumoral model (PC-OP-Intra) in the testing cohort.

Model	ΔAUC (vs. PC-OP-Intra)	ΔSensitivity (vs. PC-OP-Intra)	ΔSpecificity (vs. PC-OP-Intra)	ΔAccuracy (vs. PC-OP-Intra)
PC-OP-Intra	0	0	0	0
PC-BB-DE-Peri (2mm)	+0.019	-0.102	+0.154	+0.058
PC-OP-DE-Peri (2mm)	+0.020	-0.102	+0.170	+0.067
PC-OP-FLF-Peri (2mm)	+0.006	-0.025	+0.062	+0.029
GRAY-BB-DE-Peri (2mm)	+0.007	-0.051	+0.108	+0.048
GRAY-OP-DE-Peri (2mm)	+0.019	+0.026	+0.031	+0.029
GRAY-OP-FLF-Peri (2mm)	0	-0.179	+0.154	+0.029
PC-BB-DE-Peri (4mm)	+0.007	-0.051	+0.154	+0.077
PC-OP-DE-Peri (4mm)	+0.030	-0.077	+0.154	+0.067
PC-OP-FLF-Peri (4mm)	+0.010	+0.052	-0.015	+0.010
GRAY-BB-DE-Peri (4mm)	-0.003	+0.103	-0.030	+0.019
GRAY-OP-DE-Peri (4mm)	+0.027	+0.128	-0.092	-0.010
GRAY-OP-FLF-Peri (4mm)	+0.001	-0.128	+0.093	+0.010
PC-BB-DE-Peri (6mm)	-0.030	-0.051	+0.093	+0.038
PC-OP-DE-Peri (6mm)	+0.019	-0.025	+0.047	+0.019
PC-OP-FLF-Peri (6mm)	-0.001	+0.077	-0.061	-0.010
GRAY-BB-DE-Peri (6mm)	-0.050	-0.128	+0.062	-0.010
GRAY-OP-DE-Peri (6mm)	+0.008	+0.077	-0.030	+0.010
GRAY-OP-FLF-Peri (6mm)	-0.021	-0.025	-0.046	-0.038
PC-BB-DE-Peri (8mm)	-0.042	+0.103	-0.153	-0.058
PC-OP-DE-Peri (8mm)	-0.026	+0.052	-0.123	-0.058
PC-OP-FLF-Peri (8mm)	-0.037	+0.154	-0.138	-0.029
GRAY-BB-DE-Peri (8mm)	-0.044	+0.026	-0.061	-0.029
GRAY-OP-DE-Peri (8mm)	-0.041	+0.026	-0.076	-0.038
GRAY-OP-FLF-Peri (8mm)	-0.047	+0.026	-0.138	-0.077
PC-BB-DE-Peri (10mm)	-0.012	+0.128	-0.092	-0.010
PC-OP-DE-Peri (10mm)	-0.003	-0.102	+0.108	+0.029
PC-OP-FLF-Peri (10mm)	-0.026	+0.205	-0.200	-0.048
GRAY-BB-DE-Peri (10mm)	-0.033	+0.026	-0.015	0
GRAY-OP-DE-Peri (10mm)	-0.027	+0.052	-0.046	-0.010
GRAY-OP-FLF-Peri (10mm)	-0.030	0	-0.076	-0.048

AUC, area under the curve; ΔAUC, ΔSensitivity, ΔSpecificity, and ΔAccuracy represent the differences in AUC, sensitivity, specificity, and accuracy, respectively, relative to the best intratumoral model (PC-OP-Intra). PC, pseudo-color; BB, bounding box; OP, original precise; GRAY, grayscale; DE, direct extension; Peri, peritumoral; FLF, feature-level fusion.

### Color selection for CEUS images (PC images vs. GRAY images)

Models retaining PC images outperformed their corresponding GRAY image models under the same ROI shape and intratumoral and peritumoral region fusion strategy. In the OP-DE-Peri (4mm) model, the AUC of PC images (0.837) surpassed that of GRAY images (0.834) in the testing cohort. Similarly, in the BB-DE-Peri (2mm) model, the AUC of the PC images (0.826) was higher than that of the GRAY images (0.814) in the testing cohort. These results suggest that retaining PC information can enhance the diagnostic performance of DLR models.

### ROI shape selection (OP ROI vs. BB ROI)

Models using the OP ROI outperformed those using the BB ROI when the image color and intratumoral and peritumoral region fusion strategies were identical. In the PC-DE-Peri (4mm) model, the AUC of the OP ROI was 0.837, higher than that of the BB ROI (0.814) in the testing cohort. Similarly, in the GRAY-DE-Peri (4mm) model, the AUC of the OP ROI was 0.834, surpassing the BB ROI (0.804) in the testing cohort. These results suggest that the OP ROI can capture lesion features more effectively, thereby enhancing the diagnostic efficiency of the model.

### Intratumoral and peritumoral region fusion strategy (DE strategy vs. FLF strategy)

The DE strategy outperformed the FLF strategy under identical image color and ROI shape conditions. In the PC-OP-Peri (4mm) model, the AUC of the DE model was 0.837, higher than that of the FLF model (0.817) in the testing cohort. Similarly, in the GRAY-OP-Peri (4mm) model, the AUC of the DE model was 0.834, surpassing the FLF model (0.808) in the testing cohort. These results suggest that the DE strategy can enhance the diagnostic performance of the model more effectively.

### Model interpretation

**Figure 5:**
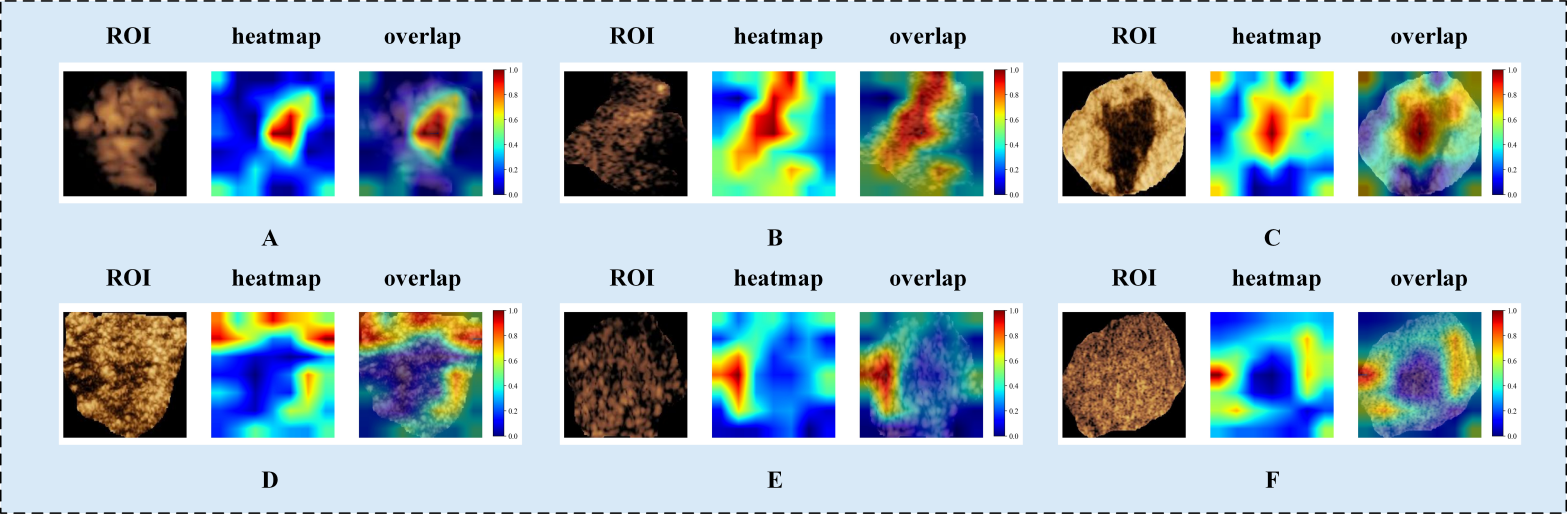
Visualized examples from different patients (labeled **A–F**). Each example includes heatmaps corresponding to CEUS images. The red regions represent a larger weight, as decoded by the color bar on the right. As shown in the figure, both the intratumoral and peritumoral regions of the tumor exhibit highlighted areas. This indicates that both the intratumoral and peritumoral regions are of significant value in the diagnosis of benign and malignant tumors. Labels **(A–F)** denote different patients and are shown for illustrative purposes only. CEUS, contrast-enhanced ultrasound; ROI, region of interest.

To explore DLR model interpretability, we used Grad-CAM to generate localization heatmaps highlighting key classification regions. We observed that both tumor boundaries and interiors are important for predicting benign versus malignant breast masses (**Figure 5**). This finding supports the value of our intratumoral-peritumoral region fusion models.

### Comparison of the best fusion model with radiologists

**Figure 6:**
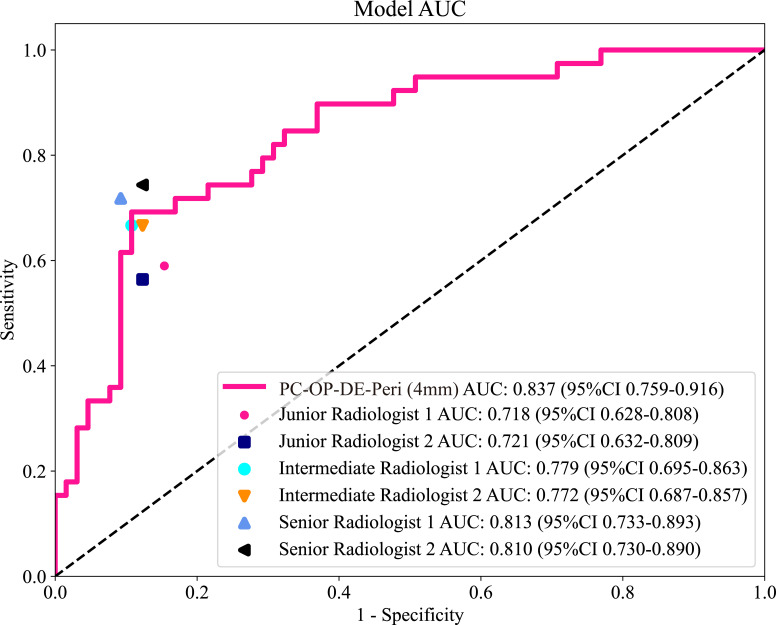
The ROC curves of six radiologists and the best intratumoral-peritumoral region fusion model in the testing cohort. AUC, the area under the curve; ROC, receiver operating characteristic; PC, pseudo-color; OP, original precise; DE, direct extension; Peri, peritumoral regions.

A reader study was performed on the testing cohort. The best intratumoral-peritumoral region fusion model, namely the PC-OP-DE-Peri (4mm) model, achieved an AUC of 0.837 in the testing cohort. To further assess the clinical applicability of the model, we compared its diagnostic performance with that of six radiologists (**Figure 6**). The results showed that the AUC of the best fusion model was significantly superior to that of two junior radiologists (*P* < 0.05), and surpassed that of intermediate and senior radiologists (*P* > 0.05). The AUCs of the six radiologists were as follows: Junior Radiologist 1 (0.718), Junior Radiologist 2 (0.721), Intermediate Radiologist 1 (0.779), Intermediate Radiologist 2 (0.772), Senior Radiologist 1 (0.813), and Senior Radiologist 2 (0.810). **Table 5** summarizes the AUC, accuracy, sensitivity, and specificity of the best fusion model and the six radiologists in the testing cohort.

**Table 5:** The comparison of diagnostic performance between the best fusion model and six radiologists.

Model	Cohort	AUC	Accuracy	Sensitivity	Specificity
PC-OP-DE-Peri (4mm)	Testing	0.837 (95%CI 0.759-0.916)	0.702	0.897	0.585
Junior Radiologist 1	Testing	0.718 (95%CI 0.628-0.808)	0.750	0.590	0.846
Junior Radiologist 2	Testing	0.721 (95%CI 0.632-0.809)	0.760	0.564	0.877
Intermediate Radiologist 1	Testing	0.779 (95%CI 0.695-0.864)	0.808	0.667	0.892
Intermediate Radiologist 2	Testing	0.772 (95%CI 0.687-0.857)	0.798	0.667	0.877
Senior Radiologist 1	Testing	0.813 (95%CI 0.733-0.893)	0.837	0.718	0.908
Senior Radiologist 2	Testing	0.810 (95%CI 0.730-0.891)	0.827	0.744	0.877

AUC, area under the curve; 95%CI, 95% confidence interval; PC, pseudo-color; BB, bounding box; OP, original precise; GRAY, grayscale; DE, direct extension; Peri, peritumoral; FLF, feature-level fusion.

## Discussion

This is the first study to systematically analyze the impact of different combinations of key parameters on the diagnostic performance of intratumoral-peritumoral region fusion models. We evaluated the influence of CEUS image color, ROI shape, and the fusion strategy of intratumoral and peritumoral regions on the diagnostic efficacy of DLR models. The results revealed the optimal combination of these parameters, thus providing a novel methodological basis for optimizing DLR for BC diagnosis.

In this study, the peritumoral regions were obtained by expanding the intratumoral ROI outward by increments of 2mm, 4mm, 6mm, 8mm, and 10mm. The diagnostic performance varied significantly when different peritumoral regions were combined with the intratumoral regions to construct fusion models. The study demonstrated that the AUC of most fusion models improved compared to the intratumoral model when the peritumoral region was expanded by 2mm or 4mm. However, models with expansions of 6mm, 8mm, or 10mm generally exhibited lower AUC values. These result highlights the importance of the peritumoral region in tumor diagnosis and suggests that the size of the peritumoral region affects the predictive ability of the models. The tumor microenvironment contains various critical biological factors from a biological perspective, such as angiogenesis, lymphatic invasion, and immune cell infiltration. These factors significantly impact tumor aggressiveness, metastasis, and recurrence risk. Compared to the intratumoral region, the peritumoral region provides more valuable insights into the interaction between the tumor and surrounding tissues. Therefore, fusing the intratumoral and peritumoral regions allows for a more comprehensive capture of the tumor’s clinical features. This, in turn, enhances the diagnostic capability of the models. This conclusion aligns with previous research. Multiple studies have demonstrated that information from the peritumoral region plays a crucial role in tumor prediction (22–24).

Although the 4mm peritumoral margin was chosen based on empirical model performance, no biological or pathological rationale currently supports its universal application. Previous radiomics studies have shown diagnostic benefits from including peritumoral features but lacked histopathological justification for specific margins. For example, Xu et al. and Yu et al. reported improved prediction of lymphovascular invasion and promoter methylation status by including peritumoral regions, though they did not correlate imaging features with tissue characteristics (39, 40). These findings, together with our results, suggest the peritumoral zone carries complementary diagnostic information. However, further studies linking histopathologic markers are needed. These markers include stromal composition, microvessel density, and immune cell infiltration. Such research will help clarify the biological basis for the optimal peritumoral margin.

The improvement in fusion model performance is not only influenced by the size of the peritumoral region but also by the selection of key parameters. In this study, six groups of fusion models were constructed with different parameter settings. The results indicated that the degree of performance improvement varied depending on the choice of these parameters.

In CEUS image color selection, we found that models using PC images outperformed those using GRAY images. PC images enhance contrast and visibility by encoding varying signal intensities with colors. This is particularly beneficial in low-contrast regions, where they better highlight the differences between the tumor and surrounding tissues. In contrast, GRAY images may lose these details, especially in areas with weaker signals, leading to an insufficient representation of lesion characteristics. Overall, PC images offer more detailed information, particularly regarding subtle changes in the tumor area, giving them an advantage over GRAY images.

In the selection of ROI shape, we found that models using the OP ROI outperformed those using the BB ROI. The OP ROI minimizes interference from background noise and non-tumor regions by accurately delineating the tumor boundaries. This enables the model to focus on the tumor region and extract more precise tumor features. In contrast, the BB ROI includes both the tumor and surrounding background areas within the smallest enclosing rectangle. The background potentially introduces irrelevant regions, distracting the model during the learning process and hindering the capture of key tumor features. Moreover, the BB ROI may not align well with the complex boundaries of irregularly shaped tumors, leading to a loss of critical boundary details. The OP ROI, on the other hand, maximizes the retention of tumor boundary features, thereby improving diagnostic accuracy.

In the selection of the intratumoral and peritumoral region fusion strategy, we found that the DE strategy outperforms the FLF strategy. This suggests that the DE strategy more effectively fuses information from the intratumoral and peritumoral regions. By combining these regions at the image level, the spatial relationship between the tumor and its surrounding microenvironment is preserved. This is particularly advantageous when tumor morphology is irregular or boundaries are unclear. In contrast, the FLF strategy first extracts features separately from the intratumoral and peritumoral regions. These features are then fused, which may lead to information redundancy, particularly when complex relationships exist between these regions. Moreover, the DE strategy operates at the pixel level, directly capturing dynamic changes and spatial information of both the tumor and surrounding tissues. DE strategy enhances the modal’s predictive ability and stability, particularly in cases with irregular tumor characteristics.

Although variations in key parameter settings among models in this study led to differences in diagnostic performance, these model construction strategies remain effective. Multiple studies have demonstrated the validity of different paraments. Xu et al. (10) constructed a DLR model using CEUS PC images for BC diagnosis. Tong et al. (29) successfully differentiated pancreatic cancer from chronic pancreatitis using GRAY CEUS images. Han et al. (30) predicted occult lymph node metastasis in tongue cancer using OP ROIs. Jiang et al. (7) predicted pathological response after neoadjuvant chemotherapy for BC using BB ROIs. Wei et al. (32) predicted the risk of microvascular invasion in hepatocellular carcinoma using a DE strategy. Can et al. (33) predicted KRAS gene mutations in rectal cancer using an FLF strategy. Diagnostic performance is still influenced by the choice of key parameters while the effectiveness of these models has been established. Therefore, optimizing the combination of these parameters is expected to further enhance model performance. This study not only contributes to refining image processing strategies but also provides valuable guidance on selecting the most appropriate imaging parameters for clinical practice. Though focused on CEUS, the dual-region fusion framework can extend to multimodal imaging. BC diagnosis often uses various imaging methods. Future studies should integrate B-mode ultrasound, mammography, or MRI. This could improve diagnostic accuracy and generalizability.

The interpretation of ultrasound images is inherently subjective, and diagnostic outcomes are often significantly influenced by the radiologist’s experience (41). This subjectivity arises from the limitations of visual information recognition. It is particularly evident in cases where imaging appearances resemble one another, yet the underlying pathological mechanisms differ. For instance, the CEUS image features of inflammatory breast diseases and malignant tumors can appear similar, potentially leading to the misdiagnosis of BC (42). The reader study results of this research further support this observation. The AUC values for the six radiologists ranged from 0.718 to 0.813, indicating that greater experience tends to improve diagnostic performance, though discrepancies still exist. In contrast, the PC-OP-DE-Peri (4mm) model (AUC=0.837) demonstrated higher diagnostic performance. The result highlights the potential of DLR models in minimizing the impact of human subjectivity.

From the perspective of the characteristics of DLR models, DLR offers significant advantages in multiple areas. First, DLR models can automatically extract subtle lesions from large datasets and image features, identifying details that are often difficult for the human eye to detect. This enables DLR models to outperform human radiologists at the level of detail in diagnostic performance. Second, DLR models maintain a high degree of consistency, reducing diagnostic variability. This consistency helps mitigate factors such as workload, emotional fluctuations, or fatigue, which may affect radiologists handling large numbers of cases. As a result, DLR models provide efficient, stable, and reliable diagnostic support. This study demonstrates that the intratumoral-peritumoral region fusion model effectively addresses the limitations of radiologists in visual recognition, offering robust support for the precise diagnosis of breast tumors. Although the proposed model showed promising results, it was developed without stratified training by tumor size or BI-RADS categories. Lesion size may affect the importance of the peritumoral region. Therefore, the current fixed 4mm expansion may not apply well to all clinical cases. Future studies should include subgroup analyses. These should focus on tumor size categories: small (<10mm), medium (10–20 mm), and large (>20mm). This will help improve the model’s clinical relevance. At the same time, real-time interpretability and processing time were not formally evaluated. However, the model’s lightweight design suggests it could be deployed in real time. Future studies will focus on optimizing efficiency and adding interpretability tools to aid clinical translation.

This study has several limitations. First, due to the retrospective design, selection bias may be present. Second, the “black box” nature of DLR models presents interpretability challenges, which may hinder their clinical application in the medical field. Additionally, the external sample size was limited. Larger, multicenter studies are needed to validate the model’s generalizability. Future prospective research should aim to improve model transparency, interpretability, and optimize experimental design to strengthen validation.

In summary, this study developed intratumoral-peritumoral region fusion models based on DLR and CEUS images for the non-invasive identification of benign and malignant breast lesions. The study systematically evaluated the selection of key parameters in intratumoral-peritumoral region fusion modal construction. These included the preference for PC images over GRAY images, the superiority of OP ROIs over BB ROIs, and the advantage of the DE strategy over the FLF strategy.

## Data Availability

The raw data supporting the conclusions of this article will be made available by the authors, without undue reservation.
